# Subject Index Volume 6

**DOI:** 10.1111/jdi.12435

**Published:** 2015-10-27

**Table:** 

3-hydroxybutyrate
effects of a sodium glucose co-transporter 2 selective inhibitor, ipragliflozin, on the diurnal profile of plasma glucose in patients with type 2 diabetes: a study using continuous glucose monitoring, Yamada 699–707
acarbose
combined use of basal insulin analog and acarbose reduces postprandial glucose in patients with uncontrolled type 2 diabetes, Kim 219–226
add-on therapy
efficacy and safety of luseogliflozin added to various oral antidiabetic drugs in Japanese patients with type 2 diabetes mellitus, Seino 443–453
adenosine triphosphate-sensitive K+ channel
fructose induces glucose-dependent insulinotropic polypeptide, glucagon-like peptide-1 and insulin secretion: role of adenosine triphosphate-sensitive K+ channels, Seino 522–526
adiponectin
gene–environment interaction between adiponectin gene polymorphisms and environmental factors on the risk of diabetic retinopathy, Li 56–66
association between the level of circulating adiponectin and prediabetes: a meta-analysis, Lai 416–429
aging
therapeutic efficacy of bone marrow-derived mononuclear cells in diabetic polyneuropathy is impaired with aging or diabetes, Kondo 140–149
albuminuria
a new Classification of Diabetic Nephropathy 2014: a report from Joint Committee on Diabetic Nephropathy, Haneda 242–246
alpha-glucosidase inhibitor
efficacy and safety of the dipeptidyl peptidase-4 inhibitor sitagliptin compared with alpha-glucosidase inhibitor in Japanese patients with type 2 diabetes inadequately controlled on metformin or pioglitazone alone (Study for an Ultimate Combination Therapy to Control Diabetes with Sitagliptin-1): a multicenter, randomized, open-label, non-inferiority trial, Yokoh 182–191
amino acids
association between insulin resistance and plasma amino acid profile in non-diabetic Japanese subjects, Yamada 408–415
apolipoprotein C-III
lack of predictive power of plasma lipids or lipoproteins for gestational diabetes mellitus in Japanese women, Iimura 640–646
approximate entropy
cardiovascular autonomic function analysis using approximate entropy from 24-h heart rate variability and its frequency components in patients with type 2 diabetes, Li 227–235
asian
incretin physiology and pathophysiology from an Asian perspective, Cho 495–507
asians
efficacy and safety of linagliptin monotherapy in Asian patients with inadequately controlled type 2 diabetes mellitus: a multinational, 24-week, randomized, clinical trial, Chen 692–698
atherosclerosis
ultrasound analysis of gray-scale median value of carotid plaques is a useful reference index for cerebro-cardiovascular events in patients with type 2 diabetes, Ariyoshi 91–97
autophagy
new mechanisms of metformin action: focusing on mitochondria and the gut, Hur 600–609
bangladeshi population
comparison of the prevalence of metabolic syndrome and its association with diabetes and cardiovascular disease in the rural population of Bangladesh using the modified National Cholesterol Education Program Expert Panel Adult Treatment Panel III and International Diabetes Federation definitions, Bhowmik 280–288
behavioral or psychological risk factors
simple self-reported behavioral or psychological characteristics as risk factors for future type 2 diabetes in Japanese individuals: toranomon Hospital Health Management Center Study 14, Heianza 236–241
birthweight
association of birthweight with diabetes and insulin sensitivity or secretion in the Japanese general population, Oya 430–435
body mass index trajectory
trajectory of body mass index before the development of type 2 diabetes in Japanese men: toranomon Hospital Health Management Center Study 15, Heianza 289–294
canagliflozin
efficacy and safety of canagliflozin alone or as add-on to other oral antihyperglycemic drugs in Japanese patients with type 2 diabetes: a 52-week open-label study, Inagaki 210–218
cardiovascular autonomic function
cardiovascular autonomic function analysis using approximate entropy from 24-h heart rate variability and its frequency components in patients with type 2 diabetes, Li 227–235
*CAPN10*
detection of *CAPN10* copy number variation in Thai patients with type 2 diabetes by denaturing high performance liquid chromatography and real-time quantitative polymerase chain reaction, Plengvidhya 632–639
cell proliferation
present status of clinical deployment of glucokinase activators, Nakamura 124–132
cell type specific
cell biology of diabetic nephropathy: roles of endothelial cells, tubulointerstitial cells and podocytes, Maezawa 3–15
central obesity
regardless of central obesity, metabolic syndrome is a significant predictor of type 2 diabetes in Japanese Americans, Sakashita 527–532
children
efficacy and safety of switching to insulin glulisine from other rapid-acting insulin analogs in children with type 1 diabetes, Urakami 87–90
chromium malate
effects of chromium malate on glycometabolism, glycometabolism-related enzyme levels and lipid metabolism in type 2 diabetic rats: a dose–response and curative effects study, Feng 396–407
circulating progenitor cell
decreased levels of circulating CD34+ cells are associated with coronary heart disease in Japanese patients with type 2 diabetes, Makino 473–478
clinical practice guidelines
current status of glucose, blood pressure and lipid management in type 2 diabetes clinic attendees in Isfahan, Iran, Janghorbani 716–725
cohort studies
strength training and risk of type 2 diabetes in a Japanese working population: a cohort study, Kuwahara 655–661
combination drug therapy
efficacy and safety of the dipeptidyl peptidase-4 inhibitor sitagliptin compared with alpha-glucosidase inhibitor in Japanese patients with type 2 diabetes inadequately controlled on metformin or pioglitazone alone (Study for an Ultimate Combination Therapy to Control Diabetes with Sitagliptin-1): a multicenter, randomized, open-label, non-inferiority trial, Yokoh 182–191
Commentary
enzymatic and non-enzymatic post-translational modifications linking diabetes and heart disease, Yamamoto 16–17
can nerve conduction studies detect earlier and predict clinical diabetic neuropathy?, Jin 18–20
curious relationship between cognitive impairment and diabetic retinopathy, Kawamura 21–23
intracellular free fatty acid upholds β-cell glucose competence: the role of peroxisome proliferator-activated receptor δ and mitochondrial metabolism, Takei 133–136
mitochondrial reactive oxygen species in the pathogenesis of early diabetic nephropathy, Nishikawa 137–139
possible new therapeutic approach for obesity-related diseases: role of adiponectin receptor agonists, Lee 264–266
incretin action on bone: an added benefit?, Ma 267–268
high-throughput screening of small interfering ribonucleic acid identifies important modulators in islet dysfunction and apoptosis, Nemoto 390–392
*quo vadis*: where have the β-cells gone?, Yagihashi 393–395
metformin as an anticancer drug: a Commentary on the metabolic determinants of cancer cell sensitivity to glucose limitation and biguanides, Song 516–518
epidermal growth factor receptor signaling and the progression of diabetic nephropathy, Koya 519–521
pharmacological interventions for painful diabetic neuropathy: comparative analysis using network meta-analysis, Yasuda 620–621
sarcopenia and diabetes: hyperglycemia is a risk factor for age-associated muscle mass and functional reduction, Umegaki 623–624
conditional targeting
cell biology of diabetic nephropathy: roles of endothelial cells, tubulointerstitial cells and podocytes, Maezawa 3–15
continuous glucose monitoring
effects of a sodium glucose co-transporter 2 selective inhibitor, ipragliflozin, on the diurnal profile of plasma glucose in patients with type 2 diabetes: a study using continuous glucose monitoring, Yamada 699–707
continuous subcutaneous insulin infusion
the relationship between the frequency of self-monitoring of blood glucose and glycemic control in patients with type 1 diabetes mellitus on continuous subcutaneous insulin infusion or on multiple daily injections, Murata 687–691
copy number variations
detection of *CAPN10* copy number variation in Thai patients with type 2 diabetes by denaturing high performance liquid chromatography and real-time quantitative polymerase chain reaction, Plengvidhya 632–639
corneal C fiber pathology
morphological changes of the peripheral nerves evaluated by high-resolution ultrasonography are associated with the severity of diabetic neuropathy, but not corneal nerve fiber pathology in patients with type 2 diabetes, Ishibashi 334–342
corneal confocal microscopy
corneal confocal microscopy: recent progress in the evaluation of diabetic neuropathy, Papanas 381–389
coronary heart disease
decreased levels of circulating CD34+ cells are associated with coronary heart disease in Japanese patients with type 2 diabetes, Makino 473–478
cyclosporine
efficacy of oral glucocorticoid and cyclosporine in a case of rituximab-refractory type B insulin resistance syndrome, Takei 734–738
cytochrome P450
rapid onset of syndrome of inappropriate antidiuretic hormone secretion induced by duloxetine in an elderly type 2 diabetic patient with painful diabetic neuropathy, Kamei 343–345
degludec
safety, efficacy, and early clinical experience of insulin degludec in Japanese people with diabetes mellitus: a first-year report from Japan, Kaku 610–619
denaturing high-performance liquid chromatography
detection of *CAPN10* copy number variation in Thai patients with type 2 diabetes by denaturing high performance liquid chromatography and real-time quantitative polymerase chain reaction, Plengvidhya 632–639
diabetes
nitric oxide donors rescue diabetic nephropathy through oxidative-stress- and nitrosative-stress-mediated Wnt signaling pathways, Hsu 24–34
phosphatase and tensin homolog-induced putative kinase 1 and Parkin in diabetic heart: role of mitophagy, Tang 250–255
comparison of the prevalence of metabolic syndrome and its association with diabetes and cardiovascular disease in the rural population of Bangladesh using the modified National Cholesterol Education Program Expert Panel Adult Treatment Panel III and International Diabetes Federation definitions, Bhowmik 280–288
photoreceptors in diabetic retinopathy, Kern 371–380
association of birthweight with diabetes and insulin sensitivity or secretion in the Japanese general population, Oya 430–435
impact of population aging on trends in diabetes prevalence: a meta-regression analysis of 160,000 Japanese adults, Charvat 533–542
establishment of maturity-onset diabetes of the young-induced pluripotent stem cells from a Japanese patient, Yabe 543–547
current status of glucose, blood pressure and lipid management in type 2 diabetes clinic attendees in Isfahan, Iran, Janghorbani 716–725
see also type 1 diabetes
see also type 2 diabetes
diabetes complications
predictability of 1-h postload plasma glucose concentration: a 10-year retrospective cohort study, Kuang 647–654
diabetes control
current status of glucose, blood pressure and lipid management in type 2 diabetes clinic attendees in Isfahan, Iran, Janghorbani 716–725
diabetes conversation map
adherence to self-care behavior and glycemic effects using structured education, Yang 662–669
diabetes mellitus
evaluation of the fatty liver index as a predictor for the development of diabetes among insurance beneficiaries with prediabetes, Nishi 309–316
assessment of glycemic control in patients with type 2 diabetes mellitus treated with metformin–sulfonylurea combination: results of a multicenter, cross-sectional, observational study in Korea, Kim 317–324
correlation of serum testosterone with insulin resistance in elderly male type 2 diabetes mellitus patients with osteoporosis, Xia 548–552
safety, efficacy, and early clinical experience of insulin degludec in Japanese people with diabetes mellitus: a first-year report from Japan, Kaku 610–619
diabetes prevention
extent of weight reduction necessary for minimization of diabetes risk in Japanese men with visceral fat accumulation and glycated hemoglobin of 5.6–6.4%, Iwahashi 553–559
diabetic nephropathy
cell biology of diabetic nephropathy: roles of endothelial cells, tubulointerstitial cells and podocytes, Maezawa 3–15
senescence marker protein-30/gluconolactonase deficiency exacerbates diabetic nephropathy through tubular injury in a mouse model of type 1 diabetes, Okada 35–43
a new Classification of Diabetic Nephropathy 2014: a report from Joint Committee on Diabetic Nephropathy, Haneda 242–246
diabetic polyneuropathy
therapeutic efficacy of bone marrow-derived mononuclear cells in diabetic polyneuropathy is impaired with aging or diabetes, Kondo 140–149
corneal confocal microscopy: recent progress in the evaluation of diabetic neuropathy, Papanas 381–389
diabetic retinopathy
gene–environment interaction between adiponectin gene polymorphisms and environmental factors on the risk of diabetic retinopathy, Li 56–66
photoreceptors in diabetic retinopathy, Kern 371–380
dipeptidyl peptidase-4 inhibitor
effects of sitagliptin on ectopic fat contents and glucose metabolism in type 2 diabetic patients with fatty liver: a pilot study, Watanabe 164–172
vildagliptin vs liraglutide as a second-line therapy switched from sitagliptin-based regimens in patients with type 2 diabetes: a randomized, parallel-group study, Takeshita 192–200
comparison of sitagliptin with nateglinide on postprandial glucose and related hormones in drug-naïve Japanese patients with type 2 diabetes mellitus: a pilot study, Tanimoto 560–566
dietary carbohydrates
carbohydrate intake is associated with time spent in the euglycemic range in patients with type 1 diabetes, Ayano-Takahara 678–686
double tracer
effects of sitagliptin on ectopic fat contents and glucose metabolism in type 2 diabetic patients with fatty liver: a pilot study, Watanabe 164–172
duloxetine
rapid onset of syndrome of inappropriate antidiuretic hormone secretion induced by duloxetine in an elderly type 2 diabetic patient with painful diabetic neuropathy, Kamei 343–345
early diabetic nephropathy
renoprotective effects of atorvastatin compared with pravastatin on progression of early diabetic nephropathy, Takazakura 346–353
early onset
maternal protein restriction induces early-onset glucose intolerance and alters hepatic genes expression in the peroxisome proliferator-activated receptor pathway in offspring, Zheng 269–279
Editorial
brave new world: our journal has become an Open Access journal, Hotta 1–2
new risk factors for obesity and diabetes: environmental chemicals, Jeon 109–111
biomarkers of progression in diabetic nephropathy: the past, present and future, Lee 247–249
branched-chain amino acids and the association with type 2 diabetes, Chen 369–370
targeting inflammation in type 2 diabetes by antibody-mediated Tyro-3, Axl, Mer receptor activation, Lee 491–494
cardiovascular effects of the incretin-based therapy: the good, the bad, or the ugly?, Cho 597–599
eicosapentaenoic acid
ultrasound analysis of gray-scale median value of carotid plaques is a useful reference index for cerebro-cardiovascular events in patients with type 2 diabetes, Ariyoshi 91–97
emotional disorders
effect of peer education on self-management and psychological status in type 2 diabetes patients with emotional disorders, Liu 479–486
endothelial function
decreased levels of circulating CD34+ cells are associated with coronary heart disease in Japanese patients with type 2 diabetes, Makino 473–478
estradiol
correlation of serum testosterone with insulin resistance in elderly male type 2 diabetes mellitus patients with osteoporosis, Xia 548–552
ezetimibe
ezetimibe combined with standard diet and exercise therapy improves insulin resistance and atherosclerotic markers in patients with metabolic syndrome, Ohbu-Murayama 325–333
fasting plasma glucose
impact of hemoglobin A1c-based criterion on diagnosis of prediabetes: the Korea National Health and Nutrition Examination Survey 2011, Kim 51–55
fatty liver
effects of sitagliptin on ectopic fat contents and glucose metabolism in type 2 diabetic patients with fatty liver: a pilot study, Watanabe 164–172
fear
fear of hypoglycemia and its determinants in insulin-treated patients with type 2 diabetes mellitus, Sakane 567–570
fructose
fructose induces glucose-dependent insulinotropic polypeptide, glucagon-like peptide-1 and insulin secretion: role of adenosine triphosphate-sensitive K+ channels, Seino 522–526
gene–environment interaction
gene–environment interaction between adiponectin gene polymorphisms and environmental factors on the risk of diabetic retinopathy, Li 56–66
genetic association
association of variants in CDKN2A/2B and CDKAL1 genes with gestational insulin sensitivity and disposition in pregnant Han Chinese women, Mei 295–301
gestational diabetes mellitus
lack of predictive power of plasma lipids or lipoproteins for gestational diabetes mellitus in Japanese women, Iimura, 640–646
gestational metabolisms
association of variants in CDKN2A/2B and CDKAL1 genes with gestational insulin sensitivity and disposition in pregnant Han Chinese women, Mei 295–301
glinide
comparison of sitagliptin with nateglinide on postprandial glucose and related hormones in drug-naïve Japanese patients with type 2 diabetes mellitus: a pilot study, Tanimoto 560–566
glomerular filtration rate
a new Classification of Diabetic Nephropathy 2014: a report from Joint Committee on Diabetic Nephropathy, Haneda 242–246
glucagon
comparison of sitagliptin with nateglinide on postprandial glucose and related hormones in drug-naïve Japanese patients with type 2 diabetes mellitus: a pilot study, Tanimoto 560–566
glucagon-like peptide-1
response to the dipeptidyl peptidase-4 inhibitors in Japanese patients with type 2 diabetes might be associated with a diplotype of two single nucleotide polymorphisms on the interleukin-6 promoter region under a certain level of physical activity, Matsui 173–181
glucagon-like peptide-1 receptor
efficacy and safety of lixisenatide in Japanese patients with type 2 diabetes mellitus inadequately controlled by sulfonylurea with or without metformin: subanalysis of GetGoal-S, Onishi 201–209
glucagon-like peptide-1 receptor agonists
vildagliptin vs liraglutide as a second-line therapy switched from sitagliptin-based regimens in patients with type 2 diabetes: a randomized, parallel-group study, Takeshita 192–200
glucagon-like peptide-2
enteral supplementation with glutamine, fiber, and oligosaccharide modulates incretin and glucagon-like peptide-2 secretion, Joo 302–308
glucokinase
present status of clinical deployment of glucokinase activators, Nakamura 124–132
glucokinase activators
present status of clinical deployment of glucokinase activators, Nakamura 124–132
glucose
return of the glucoreceptor: glucose activates the glucose-sensing receptor T1R3 and facilitates metabolism in pancreatic β-cells, Kojima 256–263
glucose challenge test
lack of predictive power of plasma lipids or lipoproteins for gestational diabetes mellitus in Japanese women, Iimura, 640–646
glucose intolerance
maternal protein restriction induces early-onset glucose intolerance and alters hepatic genes expression in the peroxisome proliferator-activated receptor pathway in offspring, Zheng 269–279
glulisine
efficacy and safety of switching to insulin glulisine from other rapid-acting insulin analogs in children with type 1 diabetes, Urakami 87–90
glycated hemoglobin
glycated hemoglobin for the diagnosis of diabetes and prediabetes: diagnostic impact on obese and lean subjects, and phenotypic characterization, Incani 44–50
are late-night eating habits and sleep duration associated with glycemic control in adult type 1 diabetes patients treated with insulin pumps?, Matejko 460–464
glycemic control
impacts of the Great East Japan Earthquake on diabetic patients, Tanaka 577–586
carbohydrate intake is associated with time spent in the euglycemic range in patients with type 1 diabetes, Ayano-Takahara 678–686
glycometabolism
effects of chromium malate on glycometabolism, glycometabolism-related enzyme levels and lipid metabolism in type 2 diabetic rats: a dose–response and curative effects study, Feng 396–407
gut
new mechanisms of metformin action: focusing on mitochondria and the gut, Hur 600–609
heart
phosphatase and tensin homolog-induced putative kinase 1 and Parkin in diabetic heart: role of mitophagy, Tang 250–255
heart rate variability
cardiovascular autonomic function analysis using approximate entropy from 24-h heart rate variability and its frequency components in patients with type 2 diabetes, Li 227–235
heel bone stiffness
heel bone strength is related to lifestyle factors in Okinawan men with type 2 diabetes mellitus, Gushiken 150–157
hemoglobin A1c
impact of hemoglobin A1c-based criterion on diagnosis of prediabetes: the Korea National Health and Nutrition Examination Survey 2011, Kim 51–55
hepatic and muscle insulin resistance
liver steatosis is associated with insulin resistance in skeletal muscle rather than in the liver in Japanese patients with non-alcoholic fatty liver disease, Kato 158–163
homeostasis model assessment
association of birthweight with diabetes and insulin sensitivity or secretion in the Japanese general population, Oya 430–435
homeostasis model assessment of insulin resistance
association between insulin resistance and plasma amino acid profile in non-diabetic Japanese subjects, Yamada 408–415
hormone secretion
fructose induces glucose-dependent insulinotropic polypeptide, glucagon-like peptide-1 and insulin secretion: role of adenosine triphosphate-sensitive K+ channels, Seino 522–526
hypoglycemia
fear of hypoglycemia and its determinants in insulin-treated patients with type 2 diabetes mellitus, Sakane 567–570
incretin
enteral supplementation with glutamine, fiber, and oligosaccharide modulates incretin and glucagon-like peptide-2 secretion, Joo 302–308
incretin physiology and pathophysiology from an Asian perspective, Cho 495–507
insulin
effect and cardiovascular safety of adding rosiglitazone to insulin therapy in type 2 diabetes: a meta-analysis, Lu 78–86
fear of hypoglycemia and its determinants in insulin-treated patients with type 2 diabetes mellitus, Sakane 567–570
safety, efficacy, and early clinical experience of insulin degludec in Japanese people with diabetes mellitus: a first-year report from Japan, Kaku 610–619
insulin combined with Chinese medicine improves glycemic outcome through multiple pathways in patients with type 2 diabetes mellitus, Zhang 708–715
insulin allergy
protamine-containing insulin allergy and renal dysfunction in a patient with type 2 diabetes, Wu 591–593
insulin index
association of variants in *CDKN2A/2B* and *CDKAL1* genes with gestational insulin sensitivity and disposition in pregnant Han Chinese women, Mei 295–301
insulin-like growth factor-1
type 1 diabetes patients have lower strength in femoral bone determined by quantitative computed tomography: a cross-sectional study, Ishikawa 726–733
insulin pump therapy
are late-night eating habits and sleep duration associated with glycemic control in adult type 1 diabetes patients treated with insulin pumps?, Matejko 460–464
insulin resistance
ezetimibe combined with standard diet and exercise therapy improves insulin resistance and atherosclerotic markers in patients with metabolic syndrome, Ohbu-Murayama 325–333
association between insulin resistance and plasma amino acid profile in non-diabetic Japanese subjects, Yamada 408–415
correlation of serum testosterone with insulin resistance in elderly male type 2 diabetes mellitus patients with osteoporosis, Xia 548–552
insulin secretion
return of the glucoreceptor: glucose activates the glucose-sensing receptor T1R3 and facilitates metabolism in pancreatic β-cells, Kojima 256–263
intensive insulin therapy
stabilization of postprandial blood glucose fluctuations by addition of glucagon like polypeptide-analog administration to intensive insulin therapy, Ogawa 436–442
interleukin-6
response to the dipeptidyl peptidase-4 inhibitors in Japanese patients with type 2 diabetes might be associated with a diplotype of two single nucleotide polymorphisms on the interleukin-6 promoter region under a certain level of physical activity, Matsui 173–181
irisin
irisin levels are associated with urotensin II levels in diabetic patients, He 571–576
Japanese
efficacy and safety of lixisenatide in Japanese patients with type 2 diabetes mellitus inadequately controlled by sulfonylurea with or without metformin: subanalysis of GetGoal-S, Onishi 201–209
ketoacidosis
case of ketoacidosis by a sodium-glucose cotransporter 2 inhibitor in a diabetic patient with a low-carbohydrate diet, Hayami 587–590
Korea
clinical characteristics of metabolic syndrome in Korea, and its comparison with other Asian countries, Hong 508–515
Letter to the Editor
case of aniridia with a heterozygous *PAX6* mutation in which the glucagon response to arginine was evaluated, Osawa 105–106
case of newly onset type 1 diabetes after highly active antiretroviral therapy against HIV infection, Kamei 367–368
pseudoaldosteronism induced by Yokukansan in an elderly Japanese type 2 diabetic patient with Alzheimer's disease, Kamei 487–488
homeodomain interacting protein kinase 2 is downregulated through the peroxisome proliferator-activated receptor gamma signaling pathway in an insulin-resistant population, Xu 594–596
bending of a vertical cannula without alarm during insulin pump therapy as a cause of unexpected hyperglycemia: a Japanese issue?, Kuroda 739–740
linagliptin
efficacy and safety of linagliptin monotherapy in Asian patients with inadequately controlled type 2 diabetes mellitus: a multinational, 24-week, randomized, clinical trial, Chen 692–698
lipid metabolism
effects of chromium malate on glycometabolism, glycometabolism-related enzyme levels and lipid metabolism in type 2 diabetic rats: a dose–response and curative effects study, Feng 396–407
liraglutide
stabilization of postprandial blood glucose fluctuations by addition of glucagon like polypeptide-analog administration to intensive insulin therapy, Ogawa 436–442
liver steatosis
liver steatosis is associated with insulin resistance in skeletal muscle rather than in the liver in Japanese patients with non-alcoholic fatty liver disease, Kato 158–163
lixisenatide
efficacy and safety of lixisenatide in Japanese patients with type 2 diabetes mellitus inadequately controlled by sulfonylurea with or without metformin: subanalysis of GetGoal-S, Onishi 201–209
long-acting insulin
combined use of basal insulin analog and acarbose reduces postprandial glucose in patients with uncontrolled type 2 diabetes, Kim 219–226
low-carbohydrate diet
lower vegetable protein intake and higher dietary acid load associated with lower carbohydrate intake are risk factors for metabolic syndrome in patients with type 2 diabetes: *post-hoc* analysis of a cross-sectional study, Iwase 465–472
case of ketoacidosis by a sodium-glucose cotransporter 2 inhibitor in a diabetic patient with a low-carbohydrate diet, Hayami 587–590
luseogliflozin
efficacy and safety of luseogliflozin added to various oral antidiabetic drugs in Japanese patients with type 2 diabetes mellitus, Seino 443–453
male
heel bone strength is related to lifestyle factors in Okinawan men with type 2 diabetes mellitus, Gushiken 150–157
maltase glucoamylase
peptide modulators of alpha-glucosidase, Roskar 625–631
maternal low-protein diet
maternal protein restriction induces early-onset glucose intolerance and alters hepatic genes expression in the peroxisome proliferator-activated receptor pathway in offspring, Zheng 269–279
maturity-onset diabetes of the young
establishment of maturity-onset diabetes of the young-induced pluripotent stem cells from a Japanese patient, Yabe 543–547
meta-analysis
association between sugar-sweetened beverages and type 2 diabetes: a meta-analysis, Wang 360–366
association between the level of circulating adiponectin and prediabetes: a meta-analysis, Lai 416–429
metabolic syndrome
testosterone level in men with type 2 diabetes mellitus and related metabolic effects: a review of current evidence, Cheung 112–123
comparison of the prevalence of metabolic syndrome and its association with diabetes and cardiovascular disease in the rural population of Bangladesh using the modified National Cholesterol Education Program Expert Panel Adult Treatment Panel III and International Diabetes Federation definitions, Bhowmik 280–288
ezetimibe combined with standard diet and exercise therapy improves insulin resistance and atherosclerotic markers in patients with metabolic syndrome, Ohbu-Murayama 325–333
high-normal urinary albumin-to-creatinine ratio is independently associated with metabolic syndrome in Chinese patients with type 2 diabetes mellitus: a cross-sectional community-based study, Li 354–359
lower vegetable protein intake and higher dietary acid load associated with lower carbohydrate intake are risk factors for metabolic syndrome in patients with type 2 diabetes: post-hoc analysis of a cross-sectional study, Iwase 465–472
clinical characteristics of metabolic syndrome in Korea, and its comparison with other Asian countries, Hong 508–515
regardless of central obesity, metabolic syndrome is a significant predictor of type 2 diabetes in Japanese Americans, Sakashita 527–532
metformin
assessment of glycemic control in patients with type 2 diabetes mellitus treated with metformin–sulfonylurea combination: results of a multicenter, cross-sectional, observational study in Korea, Kim 317–324
mitochondria
new mechanisms of metformin action: focusing on mitochondria and the gut, Hur 600–609
monotherapy
efficacy and safety of linagliptin monotherapy in Asian patients with inadequately controlled type 2 diabetes mellitus: a multinational, 24-week, randomized, clinical trial, Chen 692–698
natural disaster
impacts of the Great East Japan Earthquake on diabetic patients, Tanaka 577–586
neurotrophic factors
therapeutic efficacy of bone marrow-derived mononuclear cells in diabetic polyneuropathy is impaired with aging or diabetes, Kondo 140–149
NO donor
nitric oxide donors rescue diabetic nephropathy through oxidative-stress- and nitrosative-stress-mediated Wnt signaling pathways, Hsu 24–34
non-alcoholic fatty liver
evaluation of the fatty liver index as a predictor for the development of diabetes among insurance beneficiaries with prediabetes, Nishi 309–316
non-alcoholic fatty liver disease
liver steatosis is associated with insulin resistance in skeletal muscle rather than in the liver in Japanese patients with non-alcoholic fatty liver disease, Kato 158–163
obesity
glycated hemoglobin for the diagnosis of diabetes and prediabetes: diagnostic impact on obese and lean subjects, and phenotypic characterization, Incani 44–50
oligosaccharide
enteral supplementation with glutamine, fiber, and oligosaccharide modulates incretin and glucagon-like peptide-2 secretion, Joo 302–308
oral antidiabetic drug
efficacy and safety of luseogliflozin added to various oral antidiabetic drugs in Japanese patients with type 2 diabetes mellitus, Seino 443–453
oral glucose tolerance test
predictability of 1-h postload plasma glucose concentration: a 10-year retrospective cohort study, Kuang 647–654
oxidative stress
association of cholesteryl ester transfer protein genotypes with paraoxonase-1 activity, lipid profile and oxidative stress in type 2 diabetes mellitus: a study in San Luis, Argentina, Siewert 67–77
peer education
effect of peer education on self-management and psychological status in type 2 diabetes patients with emotional disorders, Liu 479–486
peptide modulators
peptide modulators of alpha-glucosidase, Roskar 625–631
peripheral nerve ultrasonography
morphological changes of the peripheral nerves evaluated by high-resolution ultrasonography are associated with the severity of diabetic neuropathy, but not corneal nerve fiber pathology in patients with type 2 diabetes, Ishibashi 334–342
phage display
peptide modulators of alpha-glucosidase, Roskar 625–631
phosphatase and tensin homolog-induced putative kinase 1/Parkin
phosphatase and tensin homolog-induced putative kinase 1 and Parkin in diabetic heart: role of mitophagy, Tang 250–255
photoreceptors
photoreceptors in diabetic retinopathy, Kern 371–380
plaque echogenicity
ultrasound analysis of gray-scale median value of carotid plaques is a useful reference index for cerebro-cardiovascular events in patients with type 2 diabetes, Ariyoshi 91–97
pleiotropic effect
vildagliptin vs liraglutide as a second-line therapy switched from sitagliptin-based regimens in patients with type 2 diabetes: a randomized, parallel-group study, Takeshita 192–200
pluripotent stem cells
establishment of maturity-onset diabetes of the young-induced pluripotent stem cells from a Japanese patient, Yabe 543–547
polymorphism
association of cholesteryl ester transfer protein genotypes with paraoxonase-1 activity, lipid profile and oxidative stress in type 2 diabetes mellitus: a study in San Luis, Argentina, Siewert 67–77
population aging
impact of population aging on trends in diabetes prevalence: a meta-regression analysis of 160,000 Japanese adults, Charvat 533–542
postprandial blood glucose fluctuations
stabilization of postprandial blood glucose fluctuations by addition of glucagon like polypeptide-analog administration to intensive insulin therapy, Ogawa 436–442
prediabetes
impact of hemoglobin A1c-based criterion on diagnosis of prediabetes: the Korea National Health and Nutrition Examination Survey 2011, Kim 51–55
evaluation of the fatty liver index as a predictor for the development of diabetes among insurance beneficiaries with prediabetes, Nishi 309–316
association between the level of circulating adiponectin and prediabetes: a meta-analysis, Lai 416–429
prediction
trajectory of body mass index before the development of type 2 diabetes in Japanese men: toranomon Hospital Health Management Center Study 15, Heianza 289–294
prevalence
clinical characteristics of metabolic syndrome in Korea, and its comparison with other Asian countries, Hong 508–515
impact of population aging on trends in diabetes prevalence: a meta-regression analysis of 160,000 Japanese adults, Charvat 533–542
prevention
strength training and risk of type 2 diabetes in a Japanese working population: a cohort study, Kuwahara 655–661
quantitative computed tomography
type 1 diabetes patients have lower strength in femoral bone determined by quantitative computed tomography: a cross-sectional study, Ishikawa 726–733
renal dysfunction
protamine-containing insulin allergy and renal dysfunction in a patient with type 2 diabetes, Wu 591–593
renoprotective effects
renoprotective effects of atorvastatin compared with pravastatin on progression of early diabetic nephropathy, Takazakura 346–353
resistance training
strength training and risk of type 2 diabetes in a Japanese working population: a cohort study, Kuwahara 655–661
risk factors
role of elevated serum uric acid levels at the onset of overt nephropathy in the risk for renal function decline in patients with type 2 diabetes, Tanaka 98–104
risk score
simple risk score to detect rural Asian Indian (Bangladeshi) adults at high risk for type 2 diabetes, Bhowmik 670–677
rosiglitazone
effect and cardiovascular safety of adding rosiglitazone to insulin therapy in type 2 diabetes: a meta-analysis, Lu 78–86
rural Asian Indian
simple risk score to detect rural Asian Indian (Bangladeshi) adults at high risk for type 2 diabetes, Bhowmik 670–677
self-care behavior
adherence to self-care behavior and glycemic effects using structured education, Yang 662–669
self-management
effect of peer education on self-management and psychological status in type 2 diabetes patients with emotional disorders, Liu 479–486
self-monitoring of blood glucose
the relationship between the frequency of self-monitoring of blood glucose and glycemic control in patients with type 1 diabetes mellitus on continuous subcutaneous insulin infusion or on multiple daily injections, Murata 687–691
senescence marker protein-30
senescence marker protein-30/gluconolactonase deficiency exacerbates diabetic nephropathy through tubular injury in a mouse model of type 1 diabetes, Okada 35–43
Shen-Qi-Formulas
insulin combined with Chinese medicine improves glycemic outcome through multiple pathways in patients with type 2 diabetes mellitus, Zhang 708–715
single nucleotide polymorphism
response to the dipeptidyl peptidase-4 inhibitors in Japanese patients with type 2 diabetes might be associated with a diplotype of two single nucleotide polymorphisms on the interleukin-6 promoter region under a certain level of physical activity, Matsui 173–181
sitagliptin
efficacy and safety of the dipeptidyl peptidase-4 inhibitor sitagliptin compared with alpha-glucosidase inhibitor in Japanese patients with type 2 diabetes inadequately controlled on metformin or pioglitazone alone (Study for an Ultimate Combination Therapy to Control Diabetes with Sitagliptin-1): a multicenter, randomized, open-label, non-inferiority trial, Yokoh 182–191
sleep duration
are late-night eating habits and sleep duration associated with glycemic control in adult type 1 diabetes patients treated with insulin pumps?, Matejko 460–464
small fibers
corneal confocal microscopy: recent progress in the evaluation of diabetic neuropathy, Papanas 381–389
sodium–glucose cotransporter 2 inhibitor
efficacy and safety of canagliflozin alone or as add-on to other oral antihyperglycemic drugs in Japanese patients with type 2 diabetes: a 52-week open-label study, Inagaki 210–218
case of ketoacidosis by a sodium-glucose cotransporter 2 inhibitor in a diabetic patient with a low-carbohydrate diet, Hayami 587–590
effects of a sodium glucose co-transporter 2 selective inhibitor, ipragliflozin, on the diurnal profile of plasma glucose in patients with type 2 diabetes: a study using continuous glucose monitoring, Yamada 699–707
statins
renoprotective effects of atorvastatin compared with pravastatin on progression of early diabetic nephropathy, Takazakura 346–353
structured education
adherence to self-care behavior and glycemic effects using structured education, Yang 662–669
sugar-sweetened beverages
association between sugar-sweetened beverages and type 2 diabetes: a meta-analysis, Wang 360–366
sulfonylurea
assessment of glycemic control in patients with type 2 diabetes mellitus treated with metformin–sulfonylurea combination: results of a multicenter, cross-sectional, observational study in Korea, Kim 317–324
effects of sulfonylurea as initial treatment on testosterone of middle-aged men with type 2 diabetes: a 16-week, pilot study, Wong 454–459
sweet taste receptor
return of the glucoreceptor: glucose activates the glucose-sensing receptor T1R3 and facilitates metabolism in pancreatic β-cells, Kojima 256–263
sympathetic activation
impacts of the Great East Japan Earthquake on diabetic patients, Tanaka 577–586
syndrome of inappropriate antidiuretic hormone secretion
rapid onset of syndrome of inappropriate antidiuretic hormone secretion induced by duloxetine in an elderly type 2 diabetic patient with painful diabetic neuropathy, Kamei 343–345
systemic lupus erythematosus
efficacy of oral glucocorticoid and cyclosporine in a case of rituximab-refractory type B insulin resistance syndrome, Takei 734–738
testosterone
testosterone level in men with type 2 diabetes mellitus and related metabolic effects: a review of current evidence, Cheung 112–123
effects of sulfonylurea as initial treatment on testosterone of middle-aged men with type 2 diabetes: a 16-week, pilot study, Wong 454–459
tubular injury
senescence marker protein-30/gluconolactonase deficiency exacerbates diabetic nephropathy through tubular injury in a mouse model of type 1 diabetes, Okada 35–43
type 1 diabetes
efficacy and safety of switching to insulin glulisine from other rapid-acting insulin analogs in children with type 1 diabetes, Urakami 87–90
type 1 diabetes patients have lower strength in femoral bone determined by quantitative computed tomography: a cross-sectional study, Ishikawa 726–733
type 1 diabetes mellitus
carbohydrate intake is associated with time spent in the euglycemic range in patients with type 1 diabetes, Ayano-Takahara 678–686
the relationship between the frequency of self-monitoring of blood glucose and glycemic control in patients with type 1 diabetes mellitus on continuous subcutaneous insulin infusion or on multiple daily injections, Murata 687–691
type 2 diabetes
glycated hemoglobin for the diagnosis of diabetes and prediabetes: Diagnostic impact on obese and lean subjects, and phenotypic characterization, Incani 44–50
effect and cardiovascular safety of adding rosiglitazone to insulin therapy in type 2 diabetes: a meta-analysis, Lu 78–86
testosterone level in men with type 2 diabetes mellitus and related metabolic effects: a review of current evidence, Cheung 112–123
combined use of basal insulin analog and acarbose reduces postprandial glucose in patients with uncontrolled type 2 diabetes, Kim 219–226
simple self-reported behavioral or psychological characteristics as risk factors for future type 2 diabetes in Japanese individuals: toranomon Hospital Health Management Center Study 14, Heianza 236–241
trajectory of body mass index before the development of type 2 diabetes in Japanese men: toranomon Hospital Health Management Center Study 15, Heianza 289–294
morphological changes of the peripheral nerves evaluated by high-resolution ultrasonography are associated with the severity of diabetic neuropathy, but not corneal nerve fiber pathology in patients with type 2 diabetes, Ishibashi 334–342
association between sugar-sweetened beverages and type 2 diabetes: a meta-analysis, Wang 360–366
effects of sulfonylurea as initial treatment on testosterone of middle-aged men with type 2 diabetes: a 16-week, pilot study, Wong 454–459
fear of hypoglycemia and its determinants in insulin-treated patients with type 2 diabetes mellitus, Sakane 567–570
irisin levels are associated with urotensin II levels in diabetic patients, He 571–576
protamine-containing insulin allergy and renal dysfunction in a patient with type 2 diabetes, Wu 591–593
predictability of 1-h postload plasma glucose concentration: a 10-year retrospective cohort study, Kuang 647–654
simple risk score to detect rural Asian Indian (Bangladeshi) adults at high risk for type 2 diabetes, Bhowmik 670–677
insulin combined with Chinese medicine improves glycemic outcome through multiple pathways in patients with type 2 diabetes mellitus, Zhang 708–715
type 2 diabetes mellitus
association of cholesteryl ester transfer protein genotypes with paraoxonase-1 activity, lipid profile and oxidative stress in type 2 diabetes mellitus: a study in San Luis, Argentina, Siewert 67–77
heel bone strength is related to lifestyle factors in Okinawan men with type 2 diabetes mellitus, Gushiken 150–157
efficacy and safety of canagliflozin alone or as add-on to other oral antihyperglycemic drugs in Japanese patients with type 2 diabetes: a 52-week open-label study, Inagaki 210–218
high-normal urinary albumin-to-creatinine ratio is independently associated with metabolic syndrome in Chinese patients with type 2 diabetes mellitus: a cross-sectional community-based study, Li 354–359
lower vegetable protein intake and higher dietary acid load associated with lower carbohydrate intake are risk factors for metabolic syndrome in patients with type 2 diabetes: post-hoc analysis of a cross-sectional study, Iwase 465–472
incretin physiology and pathophysiology from an Asian perspective, Cho 495–507
regardless of central obesity, metabolic syndrome is a significant predictor of type 2 diabetes in Japanese Americans, Sakashita 527–532
type 2 diabetic nephropathy
role of elevated serum uric acid levels at the onset of overt nephropathy in the risk for renal function decline in patients with type 2 diabetes, Tanaka 98–104
type B insulin resistance syndrome
efficacy of oral glucocorticoid and cyclosporine in a case of rituximab-refractory type B insulin resistance syndrome, Takei 734–738
uric acid
role of elevated serum uric acid levels at the onset of overt nephropathy in the risk for renal function decline in patients with type 2 diabetes, Tanaka 98–104
urinary-to-albumin creatinine ratio
high-normal urinary albumin-to-creatinine ratio is independently associated with metabolic syndrome in Chinese patients with type 2 diabetes mellitus: a cross-sectional community-based study, Li 354–359
urotensin II
irisin levels are associated with urotensin II levels in diabetic patients, He 571–576
visceral fat accumulation
extent of weight reduction necessary for minimization of diabetes risk in Japanese men with visceral fat accumulation and glycated hemoglobin of 5.6–6.4%, Iwahashi 553–559
weight reduction
extent of weight reduction necessary for minimization of diabetes risk in Japanese men with visceral fat accumulation and glycated hemoglobin of 5.6–6.4%, Iwahashi 553–559
Wnt
nitric oxide donors rescue diabetic nephropathy through oxidative-stress- and nitrosative-stress-mediated Wnt signaling pathways, Hsu 24–34

